# Epigenetic regulation of the nuclear genome associated with mitochondrial dysfunction in Leber’s hereditary optic neuropathy (LHON)

**DOI:** 10.1038/s41439-023-00258-5

**Published:** 2024-01-25

**Authors:** Aswathy P. Nair, Ambika Selvakumar, Janani Gopalarethinam, B. Abishek Kumar, Balachandar Vellingiri, Mohana Devi Subramaniam

**Affiliations:** 1 SN ONGC Department of Genetics and Molecular Biology, Vision Research Foundation, Sankara Nethralaya, 600006 Chennai, India; 2 Department of Biotechnology, Alagappa University, 630003 Karaikudi, Tamil Nadu India; 3 Department of Neuro-Ophthalmology, Medical Research Foundation, 600006 Chennai, India; 4 Department of Zoology, School of Basic Sciences, Central University of Punjab, Bathinda, India

**Keywords:** Imprinting, Genetics research

## Abstract

Leber’s hereditary optic neuropathy (LHON) is a mitochondrial hereditary disease in which visual loss affects complex 1 activity of the electron transport chain of mitochondria. It first manifests as painless dulling or blurry in one or even both eyes, and as it develops, sharpness and color perception are lost. In addition to primary mitochondrial DNA (mtDNA) mutations, there are also other environmental and epigenetic factors involved in the pathogenesis of LHON. One of the most common locations for deadly pathogenic mutations in humans is the human complex I accessory NDUFS4 subunit gene. The iron-sulfur clusters of the electron input domain were distorted in the absence of *NDUFS4*, which reduced complex I function and elevated the production of reactive oxygen species. Therefore, here, we studied the epigenetic alterations of *NDUFS4* by focusing on histone activation and repressive markers. We isolated peripheral blood mononuclear cells (PBMCs) from LHON patients and healthy individuals and examined epigenetic modifications in *ND4* mutant cells and control cells. Chromatin immunoprecipitation-qRT PCR (ChIP-qRT PCR) assays were performed to investigate the modifications of histones. In comparison to their controls, both LHON patients and *ND4* mutant cells exhibited a significant enrichment in activation and repressive markers. This finding indicates that these modifications might mitigate the impact of LHON mutations on complex 1 and aid in elucidating the mechanism underlying the progression of LHON disease.

## Decoding LHON: How Epigenetic Alterations Shield Complex 1

This summary was initially drafted using artificial intelligence, then revised and fact-checked by the author.

Leber’s hereditary optic neuropathy is a hereditary condition causing swift vision loss due to damage of the retina (light-sensitive layer at the back of the eye) and optic nerve (transmits visual information to the brain). The role of specific changes in mitochondrial DNA (DNA located in the mitochondria, the powerhouses of cells) in this disease and the impact of environmental and genetic factors are still unclear. Scientists studied a gene called NDUFS4, essential for cells’ energy production and associated with this condition. They observed alterations in this gene in patients and cell lines with disease-inducing mutations, compared to healthy people and cells. This included examining changes in histone modifications. Significant differences were observed in these modifications between patient samples and controls, offering useful knowledge about the disease’s progression. This study could guide future treatments targeting these modifications.

## Introduction

Leber’s hereditary optic neuropathy (LHON) is a mitochondrial genetic condition with mitochondrial DNA (mtDNA) mutations in complex 1 of the electron transport chain^1^. Rapid bilateral central vision loss caused by retinal ganglion cell layer and optic nerve degeneration is a hallmark feature of Leber’s hereditary optic neuropathy (LHON)^2^. The majority of patients carry one of three primary mtDNA point mutations: m.3460 G > A (*MTND1)*, m.11778 G > A (*MTND4*), or m.14484 T > C. (*MTND6*)^3^. Given the potential impact of the mtDNA haplogroup, in India, the m.11778 G > A mutation is predominant^4^. Primary mitochondrial DNA (mtDNA) mutations are required. However, they are not sufficient to produce optic neuropathy since disease penetrance can even differ between different families that have the same mutation. Thus, in recent years, the notion that significant environmental and/or genetic differences may have an impact on the penetrance and risk of developing LHON has gained support^5^. Numerous mechanisms have been proposed for LHON, including reduced ATP generation and mitochondrial respiratory chain function, loss of membrane potential, increased formation of reactive oxygen species (ROS) and glutamate excitotoxicity^6^. Since 80–90% of cases are male, LHON predominantly affects men. The male preponderance and severe incomplete penetrance imply additional genetic and environmental variables that control the phenotypic expression of LHON. There is now convincing evidence that environmental triggers, specifically smoking, play a role in LHON. It is interesting to note that an in vitro study utilizing fibroblasts from patients suggests that smoking has a deleterious impact on cell survival by changing mtDNA copy number, oxidative phosphorylation, and ROS detoxification. The initiation of vision loss in LHON has also been connected to accidental exposure to industrial chemicals and some medications with suspected mitochondrial toxic effects, such as ethambutol and antiretroviral medicines^5^.

A large membrane protein complex called proton-pumping NADH:ubiquinone oxidoreductase (complex I) is essential for aerobic energy metabolism. Through a chain of seven iron-sulfur clusters, it oxidizes NADH using a noncovalently bound flavin and transfers the electrons to ubiquinone across a distance of approximately 100 A^0^. The enzyme helps to produce the proton motive force that powers ATP synthase by combining the movement of two electrons and four protons across the inner mitochondrial membrane^7^. Primary complex 1 abnormalities are caused by mutations in the genes that encode the structural subunits and assembly components of complex 1. The *MT-ND1, MT-ND4* and *MT-ND6* genes, which are associated with LHON disease, encode subunits in complex 1, and mutations in these mitochondrial genes reduce complex 1 activity. LHON mutations have a significant impact on mitochondrial function, particularly within complex 1, leading to impaired electron transfer, reduced ATP production and increased production of reactive oxygen species (ROS)^3^. There are a number of nuclear and mitochondrial gene mutations that have been linked to mitochondrial complex 1 impairment^8^. Cells experience energy depletion and faulty oxidative phosphorylation as a result of these alterations in mitochondrial protein levels^8^. Any minute mitochondrial abnormalities that result in mitochondrial malfunction also contribute to the pathogenesis of LHON^9^. Complex 1 has 3 modules: 1) the N module, the NADH-oxidizing electron input module; 2) the ubiquinone-reducing Q module; and 3) the proton-translocating P module^10^. All these functional modules in complex 1 should be assembled properly to create complex 1 functional holoenzymes. The P module is the hydrophobic arm of complex 1 that contains 29 protein subunits, including mitochondrial-encoded *ND1, ND4* and *ND6*. The core subunits of the N module, *NDUFV1*, *NDUFV2*, and *NDUFS1*, and the accessory subunits of the N module, *NDUFV3, NDUFS4, NDUFS6* and *NDUFA12*, are finally combined^11^. Mutations in these genes, including both nuclear-encoded and mitochondrial-encoded genes, can lead to complex 1 dysfunction and impairment of the electron transport chain, which contribute to mitochondrial diseases. Efficient assembly of complex 1 is vital for its stable and functional conformation. The intricate interplay between various subunits ensures the optimal functioning of the complex in the respiratory chain^11^. A nuclear-encoded gene called *NDUFS4* encodes a complex I matrix arm component of the N module. *NDUFS4* mutations cause substantial problems in complex I activity as well as impairments in assembly and stability^12^. *NDUFS4* appears to be involved in the latter stages of complex I assembly, according to ample evidence. Quantitative mass spectrometry analysis demonstrated that deletion of *NDUFS4* increased the levels of the assembly factor *NDUFAF2* and generated a nearly complete loss of the accessory complex I component *NDUFA12* in patient cell lines and animal models^13^. *NDUFS4* can also be distinguished because it contains a conventional serine phosphorylation site in mammals^14^. Most of the *NDUFS4* subunit mutations that have been reported result in the deletion of the *NDUFS4* phosphorylation site, which favorably regulates the NADH-ubiquinone oxidoreductase activity of the CI^15^.

Epigenetics is the study of variations in gene expression brought on by both intrinsic and extrinsic stimuli but are unrelated to changes in the DNA sequence^16^. In addition to mitochondrial mutations, epigenetic variables are crucial in the development and manifestation of the disease. Changes in the expression of the complex 1 subunit would be noticeable if nuclear genes that code for those subunits were subject to epigenetic control. The primary distinction between these two genomes is the histone protection of nuclear DNA, which is absent in mtDNA. Nuclear gene expression and protein synthesis in the cell are regulated by posttranslational histone modifications and different DNA methylations. The level of mitochondrial proteins decreases as a result of this type of epigenetic modification, which affects nuclear-encoded mitochondrial genes and culminates in mitochondrial malfunction^8^. Enzymes, including histone acetyltransferases (HATs), histone deacetylases (HDACs), and histone methyltransferases (HMTs), are responsible for the majority of histone modifications. Distinct types and sites of histone modification can have different consequences on gene expression, such as activation or repression^17^. Transcriptional activation is predominantly mediated by methylation of histone H3 on lysine 4 (H3K4), lysine 36 (H3K36), lysine 79 (H3K79), or arginine 17 (H3R17). However, transcriptional repression is frequently associated with methylation of histone H3 at lysine 9 (H3K9) and lysine 27 (H3K27) or histone H4 on lysine 20 (H4K20)^18^. The H3K27Me3 histone mark is repressive, and H3K4Me3 becomes a stronger mark for transcriptional activation. Histone acetylation occurs at lysine residues and transforms condensed chromatin into a relaxed form that is considered to increase gene transcription in general^19^. The link between histone and DNA is weakened when a lysine residue is given an acetyl group. This balances the positive charge on the lysine residue, enabling access to transcription machinery to initiate gene transcription. Thus, transcription is activated by histone acetylation through the relaxation of chromatin^20^. H3K27Ac and H3K18Ac modification levels are associated with promoter and transcription-enhancing activity. Analyzing these histone modifications in the gene can elucidate the effect of histone modification on gene expression at various histone enrichment levels^18^. There is no promising treatment that has been established yet for LHON, and many clinical trials on gene therapy and other medications for therapeutic purposes are ongoing^21,22^. Since methylation and acetylation are crucial epigenetic modifications that play fundamental roles in regulating gene expression and essential biological processes, understanding the role of these modifications provides insights into the complexities of cellular function and disease mechanisms. Methylation can either repress or activate gene expression depending on the locations, and acetylation generally promotes gene expression^19^. Understanding the histone modification differences among genes is important for determining gene expression regulation at the epigenetic level, which influences transcription, chromatin structure and cellular processes^23^. Therefore, in this study, we tried to elucidate the role of the *NDUFS4* (OMIM:602694 GenBank accession no: NM_002495 Protein accession no: O43181) nuclear encoding gene in LHON pathogenesis by analyzing the various histone modifications in LHON patients carrying *ND4* mutations and in *ND4* mutant cell lines. We used 5 different antibodies against histone acetylation and methylation, H3K18Ac, H3K27Ac, and H3K4Me3 for activation marks and H3K9Me2 and H3K27Me3 for repressive marks, in this study to determine differences in histone enrichment in *NDUFS4* in LHON. The differences in histone modification patterns between LHON conditions and normal conditions can provide insights into how epigenetic regulation is altered in the disease state.

## Materials and methods

### Cell culture and maintenance

Skin-derived *ND4* mutant fibroblasts from the LHON cohort were obtained from Dr. Patrick Yu Wai Man, Cambridge University, UK. The cells were grown in DMEM (Dulbecco’s modified Eagle’s medium) high glucose (0.45% glucose) media (Sigma Aldrich, USA) with 10% fetal bovine serum (FBS) enriched with 1% L-glutamine (Gibco, Thermo Fisher Scientific, USA), 1% nonessential amino acids (NAA, Sigma Aldrich, USA) and 100 U/ml penicillin and streptomycin (Sigma Aldrich, USA). The culture flask was incubated at 37 °C in 5% CO_2_ in a humidified environment. Skin fibroblasts were procured from ATCC and employed as a control. These cells were grown in DMEM containing 5% FBS, 1% L-glutamine, 1% NAA and 100 U/ml penicillin and streptomycin, and they were incubated at 37 °C with 5% CO_2_. When the cell confluency reached approximately 70–80%, the cells were subcultured by 0.05% trypsin digestion for cell line maintenance, and the media was replaced every seven days.

### Cell viability test

Cell viability was evaluated by the trypan blue assay. Cells were subjected to 0.05% trypsinization and incubated at 37 °C for 5 minutes. Then, the samples were centrifuged at 1200 rpm for 5 minutes. Then, 1 ml of fresh medium was added to the pellet. From this, 10 µl of cells was transferred to a 1 ml Eppendorf tube, and 10 µl of trypan blue dye was added and mixed well. The hemocytometer was loaded with 10 µl of the cell suspension, and visually, the number of live cells was counted.

### Proliferation assay

A CCK-8 assay (ab228554, Abcam, U.K.) was used to measure the proliferation of control fibroblasts and *MT-ND4* mutant cells. For the CCK-8 experiment, the cell density to OD ratio was optimized for 1×10^3^, 2×10^3^, 4×10^3^ and 6×10^3^ cells on a 96-well plate. After that, the wells were seeded with the ideal number of cells, and the OD was recorded every 24 hours for the next 96 hours. The cells were grown in 96-well plates with clear, flat bottoms, and 10 µl of the CCK-8 reagent was applied at the appropriate time. The cells were then incubated at 37 °C for two hours to measure the OD. An Epoch Microplate Spectrophotometer was used to detect the OD at a wavelength absorbance of 450 nm.

### Sample collection

For this study, LHON patients with known mutations and suspected patients who were clinically diagnosed with LHON were recruited. Patients with other ocular diseases were excluded from this study. According to the inclusion and exclusion criteria, 17 patients were recruited for this study. After genetic testing to confirm a LHON mutation, 8 patients were positive for LHON mutations. This study was conducted with 8 LHON patient and 8 control samples. Written informed consent was obtained from patients before collecting the blood. This study was approved by the Institutional Review Board (IRB) and ethics committee, and all procedures were performed in accordance with institutional guidelines and the Declaration of Helsinki.

### DNA extraction and genotyping of cells

In accordance with the protocol, 5×10^6^ cells were collected for DNA isolation using the QIAamp® DNA Mini Kit from QIAGEN. DNA was measured using a Nanodrop (Spectrophotometer ND-1000). The *MT-ND4* gene was amplified using specific primers after DNA isolation (forward primer: 5’ GCT CCC TTC CCC TAC TCA TC 3’; and reverse primer: 5’ AGG GGT CGT AAG CCT CTG TT 3’). After amplification, it was verified using electrophoresis on a 2% agarose gel, and DNA sequencing was carried out. Both 3’ to 5’ and 5’ to 3’ amplicon strands underwent cycle sequencing using the Big Dye Terminator cycle sequencing kit (Applied Biosystems, CA). DNA was then precipitated by combining 5 M sodium acetate with ethanol in a 5:1 ratio. The sequences were then examined using Applied Biosystems, Foster City, California (ABI 3500 Genetic Analyzer). Hi-Di formamide was used for high-quality sequencing. The sequence of *MT-ND4* was analyzed using a program called BioEdit Sequence Analyzer, and the target mutation was identified.

### Zygosity analysis

Restrictive digestion of the *MT-ND4* gene revealed *ND4* mutation zygosity. The restriction site in *ND4* was broken down with the aid of the enzyme NmuCI (Tsp45I). Next, 0.2 µl of NmuCI (Tsp45I) enzyme and 2 µl of buffer were added to 5 µl of the PCR product. The cells were incubated at 37 °C for 16 hours to ensure that *MT-ND4* was completely digested. After that, the product was processed through 4% Seakem® LE Agarose (Lonza, Rockland, ME) to consider the fragment size.

### Peripheral blood mononuclear cell (PBMC) isolation and chromatin immunoprecipitation (ChIP)

PBMCs were extracted from blood using Ficoll density gradient centrifugation. After centrifugation for 30 minutes at 2000 rpm, a bottom layer of erythrocytes and polymorphonuclear cells and a top portion of plasma underneath a hazy band of PBMCs were observed. Hazy PBMC bands were collected using sterile pipettes and washed two times with 1X PB. Then, they were centrifuged at 2000 rpm for 5 minutes to remove the serum and Ficoll.

The materials used for the ChIP assay were phosphate buffer saline (PBS, Gibco), 37% formaldehyde (Sigma Aldrich), protease inhibitor cocktail (PIC), 10X glycine, 2X chip sonication cell lysis buffer, ChIP sonication nuclear lysis buffer (Cell Signaling Technology), antibodies (Cell Signaling Technology) against H3K27Ac, H3K18Ac, H3K9Me2, H3K4Me3, and H3K27Me3, the positive control histone H3 (D2B12) XP rabbit mAb, the negative control normal rabbit IgG, protein G magnetic beads, binding buffer, wash buffer, and elution buffer (Cell Signaling Technology).

PBMCs and cells were cross-linked by adding 37% formaldehyde for 10 minutes, and cross-linking was stopped by adding 10X glycine for 5 minutes. After centrifugation at 1200 rpm for 5 minutes, the pellet was resuspended in 1 ml of 1X ChIP sonication cell lysis buffer + PIC and subjected to sonication (QsonicaQ800R3) at 40% amplitude, 5 seconds off and 5 seconds on, cycle for 5 minutes. Then, lysates were clarified by centrifugation at 21,000× *g* for 10 minutes at 4 °C. The DNA concentration was determined using a NanoDrop spectrophotometer (SpectraND-1000), and the DNA fragment size was detected using 1% agarose gel electrophoresis. One hundred microliters of digested chromatin was diluted in 400 µl of 1X ChIP buffer (1:4). Ten microliters of diluted sample was taken as 2% input and stored at -20 °C. To 500 µl of diluted chromatin, the respective immunoprecipitation antibodies were added, and the immunoprecipitated (IP) samples were incubated at 4 °C overnight with rotation.

**Table:** 

Antibody	Dilution
Acetyl-Histone H3 (Lys18) H3K18Ac	1:25
Acetyl-Histone H3 (Lys27) H3K27Ac	1:100
Di-Methyl-Histone H3 (Lys9) H3K9Me2	1:25
Tri-Methyl-Histone H3 (Lys4) H3K4Me3	1:50
Tri-Methyl-Histone H3 (Lys27) H3K27Me3	1:50

Then, 30 µl of protein G magnetic beads were added to each IP reaction and incubated at 4 °C for 2 hours with rotation. Protein G beads were pelleted by placing them in a magnetic separation rack until the solution became clear. Then, the cells were washed with low salt buffer thrice and high salt buffer twice. The cross-linking was then reversed by adding 1X ChIP elution buffer and kept in a water bath at 65 °C for 30 minutes with rotation. Then, the samples were centrifuged at 10,000 × g for 10 seconds. Protein G beads were pelleted by placing them in a magnetic separation rack until the solution became clear. The supernatant was removed carefully. Then, 6 µl of 5 M NaCl and 2 µl of proteinase K were added to the supernatant and the 2% input and incubated for 2 hours at 65 °C. Then, the DNA was purified and eluted using 50 µl of elution buffer. DNA was quantified using a NanoDrop spectrophotometer (ND-1000).

### Quantitative real-time PCR analysis

Quantified DNA was subjected to qRT‒PCR. Two microliters of DNA sample, 2 µl of primers, 10 µl of universal Simple Chip master mix (Cell Signaling Technology), and 6 µl of nuclease-free water were added to each well of the PCR plate. Primer sets (forward primer: ACTGGCTTGAGAACGAAGGA; reverse primer: GAGACTCCGCAAAAATGGAG) were used to selectively amplify the exon 1 region of *NDUFS4*. Initial denaturation at 95 °C for 3 minutes, denaturation at 95 °C for 15 minutes, and annealing and extension at 60 °C for 60 seconds were carried out. Melt curve analysis (SYBR Green) was carried out at 95 °C for 60 seconds, and signals obtained from each immunoprecipitation were expressed as a percent of the total input chromatin. The relative fold change of immunoprecipitation was calculated using the formula [2% x 2 ^C[T] 2% Input sample - C[T] IP Sample^].

### Statistical analysis

The nonparametric Mann–Whitney U test was used to analyze the relative differences in histone modifications between LHON patients and healthy individuals and *MT-ND4* mutant cells and control cells. Statistical significance was set at *P* < 0.05.

## Results

### Cell culture, viability test and proliferation assay

**Fig. 1:**
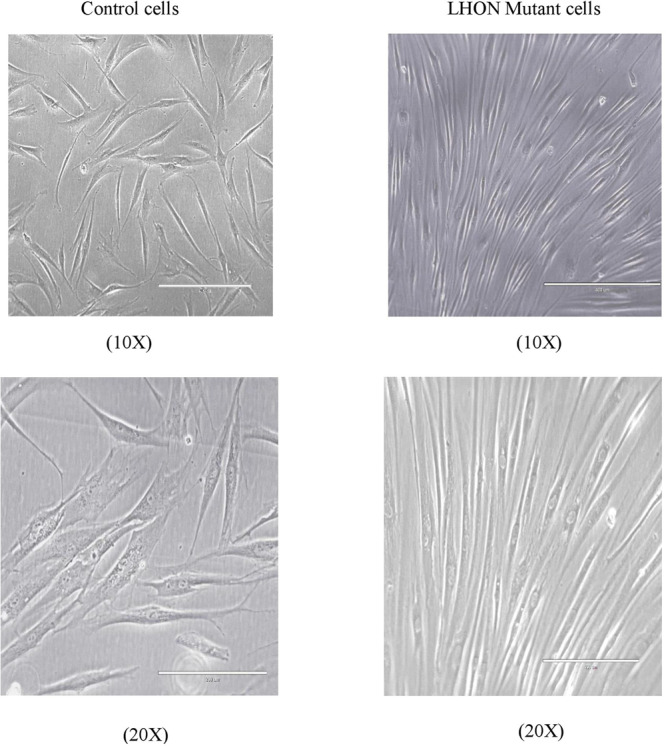
Cell culture and morphology analysis. Phase contrast images of LHON MT-ND4 mutant cells and control fibroblasts grown in their appropriate medium and conditions.

**Fig. 2:**
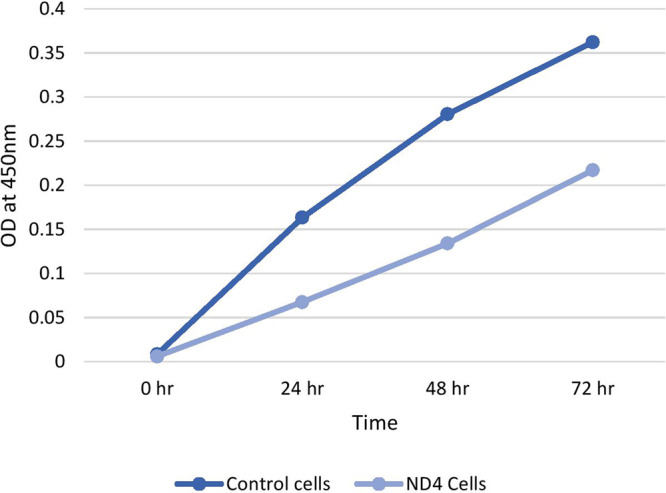
Cell proliferation assay. Proliferation study of LHON MT-ND4 mutant cells versus control cells using CCK-8 assay.

LHON *MT-ND4* mutant fibroblasts and control fibroblasts were grown in appropriate conditions. These cells were used for immunoprecipitation reactions using various antibodies against histone modifications. Figure 1 shows microscopy images of *MT-ND4* mutant cells and control cells. Cell viability was assessed by trypan blue assay, in which dead cells were stained blue and live cells were unstained. Approximately 100,000 cells were alive and maintained for future experiments. The viability and proliferation of cells were determined by a cell proliferation assay using a CCK-8 kit. The proliferation rate was analyzed using GraphPad Prism 7.0. Cell proliferation was greater in control fibroblasts than in LHON mutant cells (Fig. 2).

### Genotyping and zygosity analysis

**Fig. 3:**
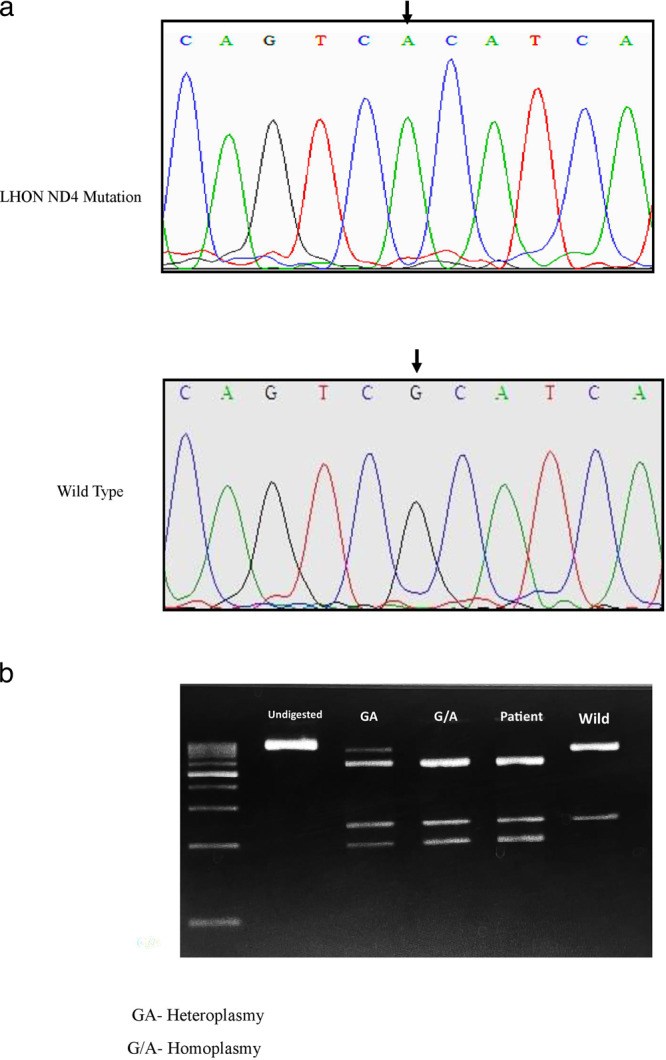
Genotyping and zygosity analysis of LHON *ND4* mutation. **a** MT-ND4 mutation analysis: Sanger sequencing electropherogram showing the G→A ND4 mutation in patients’ mitochondrial genes. **b** Restriction enzyme digestion for zygosity analysis: Restriction digestion using the enzyme NmuCI (Tsp45I) revealed that patients carried MT-ND4 homoplasmic mutations in their mitochondrial genes.

To determine LHON mutations in the mitochondrial genome, DNA from LHON patient blood was isolated, and Sanger sequencing was performed. All the individuals showed a point transition mutation (G11778A) in the *ND4* gene in the mitochondrial genome. Restriction digestion using the enzyme NmuCI (Tsp45I) was carried out to reveal the mutant zygosity. All individuals carried the *MT-ND4* (m.11778 G > A) homoplasmic mutation in their mitochondrial gene. Figure 3a shows the electropherogram results of patients carrying the *MT-ND4* point mutation **m.11778** **G** > **A** in their gene. Figure 3b shows restriction digested fragments using the enzyme NmuCI (Tsp45I), which reveals that all patients carry homoplasmic *MT-ND4* mutations.

### ChIP assay and chromatin digestion optimization

**Fig. 4:**
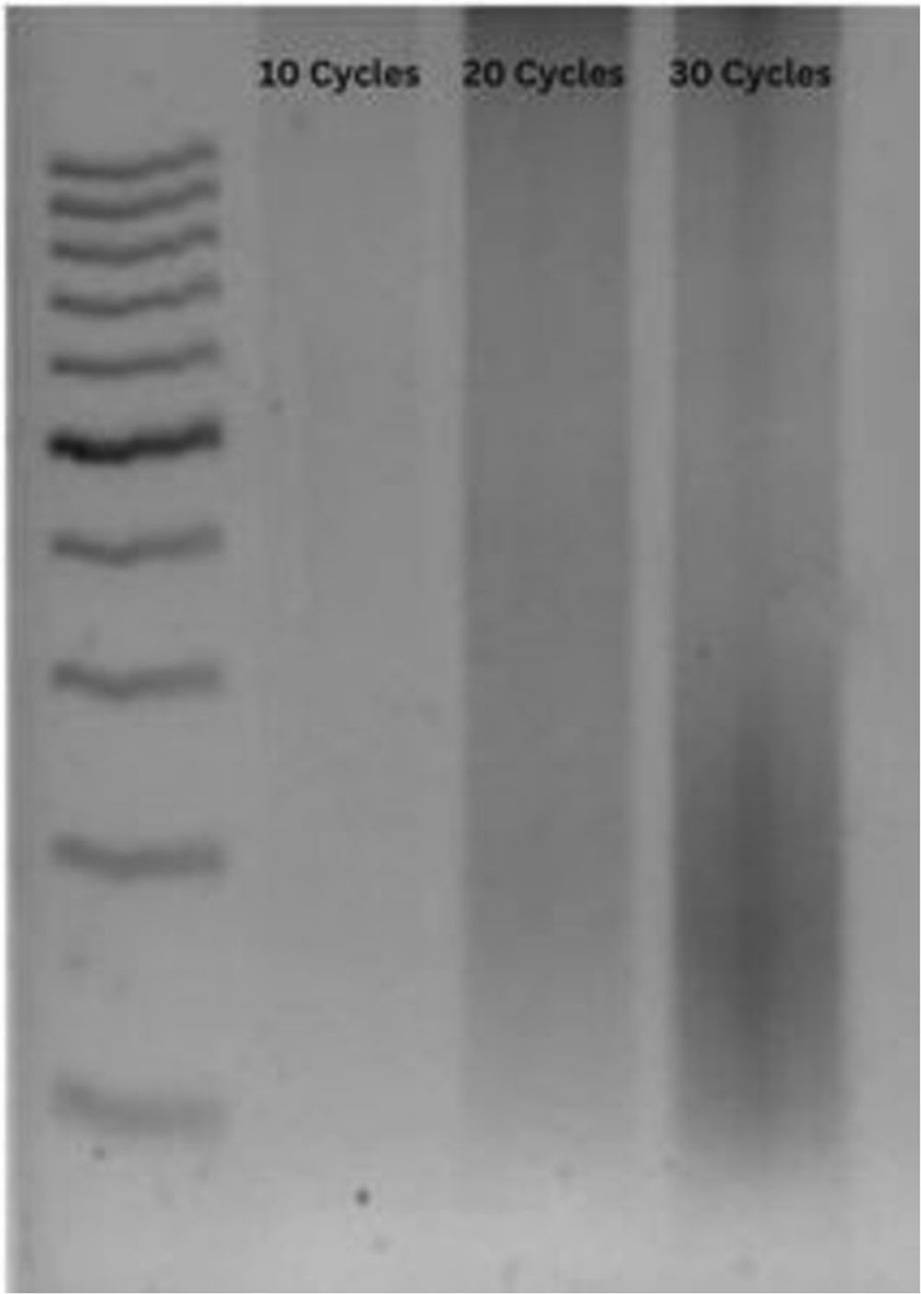
Chromatin digestion optimization for immunoprecipitation. Sonication of chromatin fragments of cells was performed by shearing at 40% amplitude for 5 minutes with 5 seconds on and 5 seconds off cycle. Sonication was optimized to 30 cycles based on the chromatin lengths in the 1% agarose gel.

We optimized a ChIP assay from PBMCs to determine the histone enrichment at the promoter region of the NDUFS4 gene in LHON patients. In the ChIP assay, chromatin digestion is one of the key steps, and optimization of the digestion conditions is crucial in determining the efficiency of the experiment. In our experiment, the sonication method was used for chromatin digestion, and sonication cycles were optimized under appropriate conditions using Qsonica Q800R3. The digested chromatin tubes were clarified by microcentrifugation at 4 °C, and DNA was isolated from a small amount of lysate to determine the accuracy of chromatin digestion. Using a NanoDrop spectrophotometer (ND-1000), the DNA was quantified, and the samples were run on a 1% agarose gel. In optimal chromatin fragmentation, the DNA smear should appear within 200 bp to 1 kb. The sonication cycle was optimized to 30 cycles based on the chromatin length. Figure 4 shows the optimization of chromatin digestion in the ChIP assay using the sonication method.

### Analysis of five histone modifications of *NDUFS4* in patient and control samples using ChIP assay

**Fig. 5:**
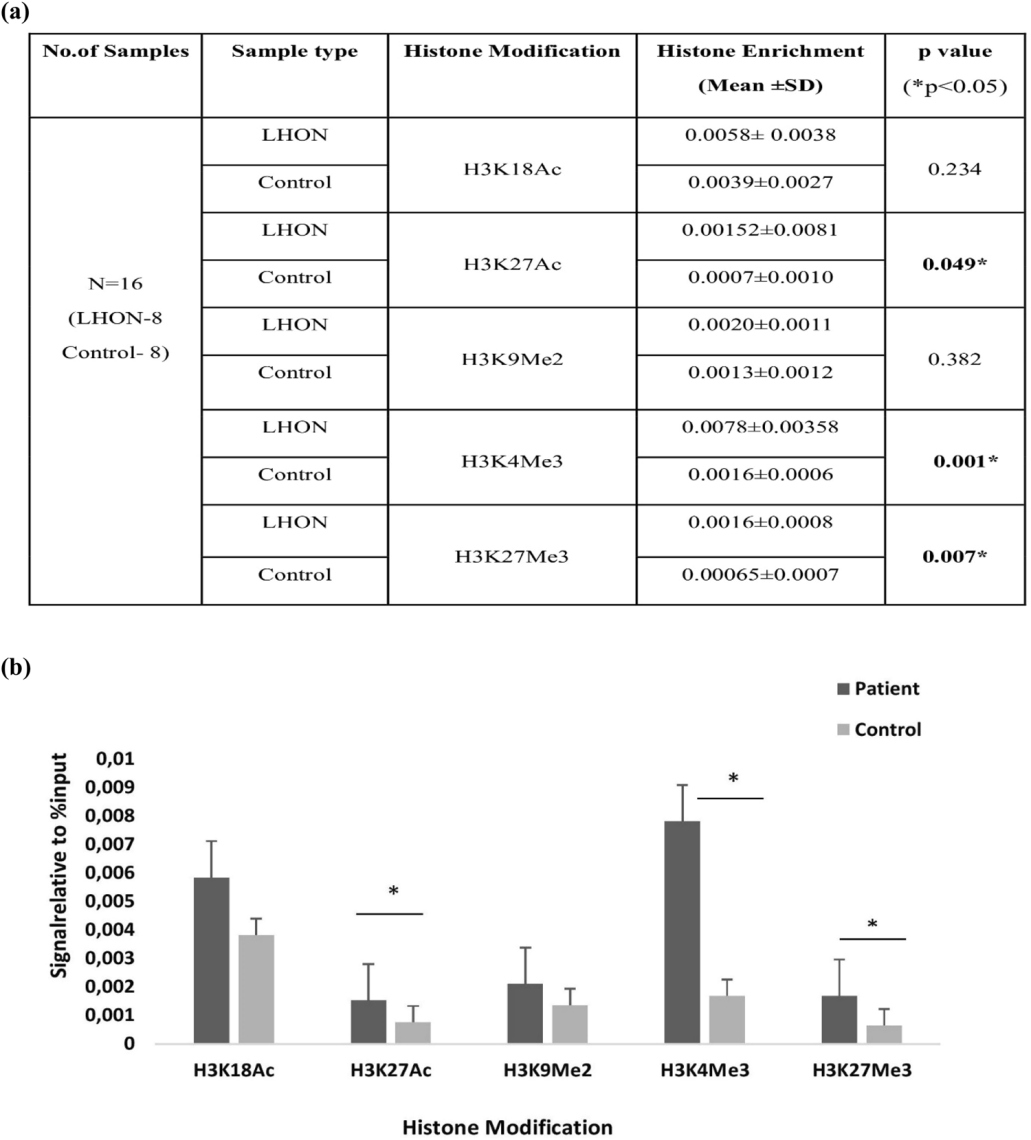
Comparative analysis of histone modifications in *NDUFS4* gene in LHON patients versus control subjects. **a** The *p* value obtained by Mann‒Whitney U-test analysis of histone modifications in the *NDUFS4* gene in LHON patients versus healthy individuals (*p < 0.05). Significant values are highlighted in bold. **b** qRT‒PCR analysis of histone modifications in the *NDUFS4* gene reveals significantly increased expression of H3K27Ac, H3K4Me3 and H3K27Me3 in LHON patients compared to control individuals (*p < 0.05).

After quantification of digested chromatin, 10 µg of chromatin was subjected to immunoprecipitation using antibodies against specific histone modifications at appropriate dilution concentrations [H3K18Ac (1:5); H3K27Ac (1 100); H3K9Me2 (1:25); H3K4Me3 (1:50); H3K27Me3 (1:50)] per the manufacturer’s instructions. Immunoprecipitated regions were pulled down using protein G magnetic beads, and cross-linking was reversed using NaCl and proteinase K treatment. DNA was purified from the decrosslinked samples. DNA was then amplified using NDUFS4 primers and analyzed by real-time PCR. The analysis determines the amount of target DNA, which is proportional to the amount of specific histone modification in *NDUFS4*. The Ct values of each immunoprecipitation reaction were normalized with their 2% input to calculate the signal generated from the specific histone modification. Histone enrichment was calculated for each histone modification in the LHON patient and control groups separately. The Mann‒Whitney U test was used to analyze the differences in histone modification enrichment between the LHON patient and control groups. Significant differences in histone modification enrichment were observed between the LHON patient and control groups (*p < 0.05) (Fig. 5a). According to previous studies, accessibility to DNA depends on the presence or absence of the concentration of histone modifications in specific loci.

In the analysis of five histone modifications, statistically significant differences in histone enrichment in H3K27Ac, H3K4Me3 and H3K27Me3 were observed between the patient and control groups (*P < 0.05). Figure 5b shows the qRT‒PCR analysis of histone modifications in *NDUFS4* in LHON patients and healthy individuals. Significant enrichment of activation and repressive markers might occur to overcome the defective function or to compensate for the effect of *MT-ND4* mutations in complex 1 functioning.

### Analysis of histone modifications of *NDUFS4* using *MT-ND4* mutant cell lines and control cells

**Fig. 6:**
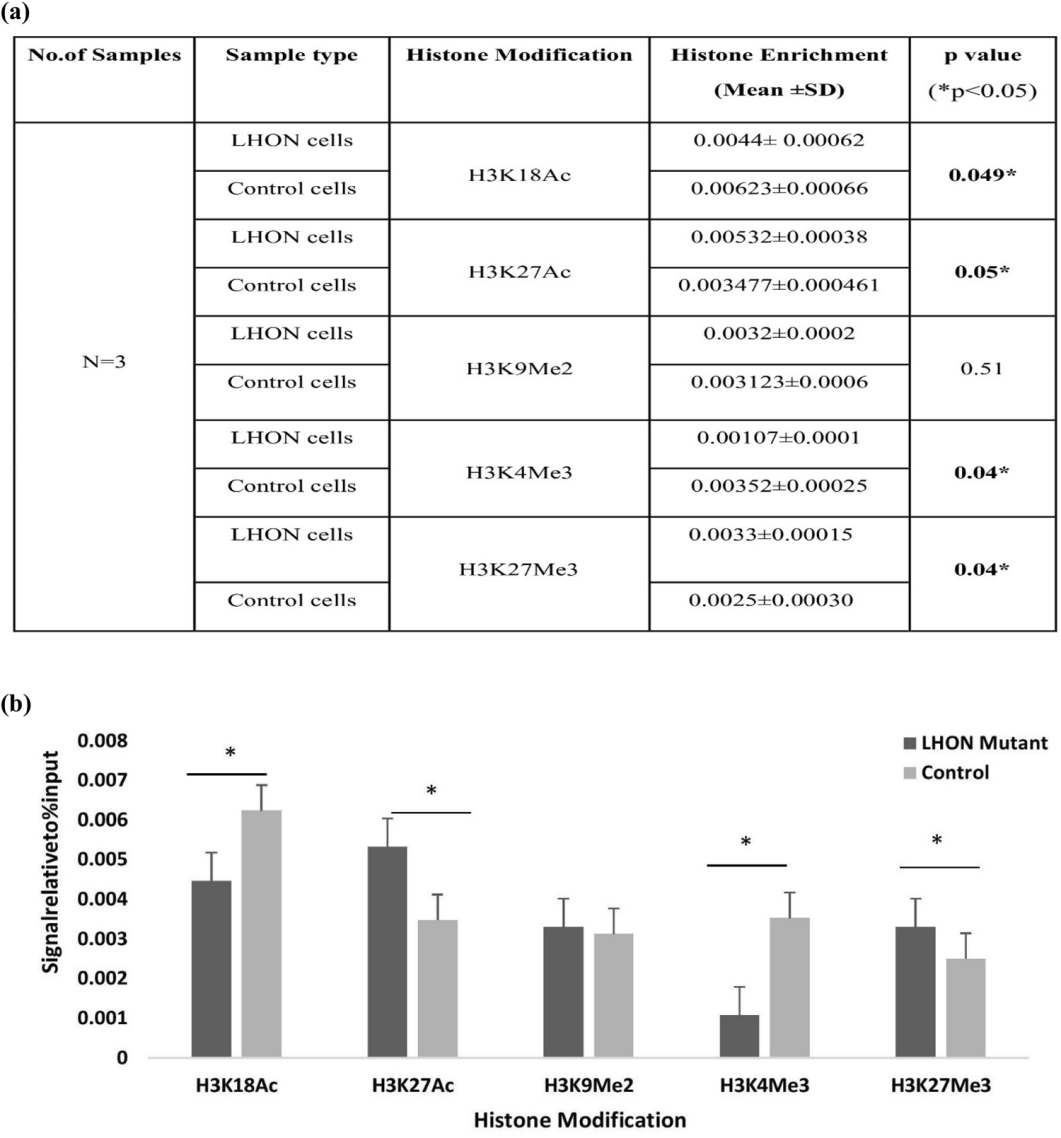
Comparative analysis of histone modifications in *NDUFS4* gene in LHON mutant cell lines versus control. **a** The *p* value obtained by Mann‒Whitney *U*-test analysis of histone modifications in the *NDUFS4* gene in LHON mutant cells versus control fibroblasts (*p < 0.05). Significant values are highlighted in bold. **b** qRT‒PCR analysis of histone modifications in the *NDUFS4* gene reveals significantly increased histone enrichment of H3K18Ac, H3K27Ac, H3K4Me3 and H3K27Me3 in *MT-ND4* mutant cells when compared to control cells (*p < 0.05).

We also conducted a ChIP assay using LHON mutant fibroblasts and control fibroblasts to examine and verify the differences in histone modifications in *NDUFS4*. For the assay, the procedure was the same as that used for the PBMCs. Immunoprecipitation was conducted using the same five different antibodies against histone modifications and was immunoprecipitated from the diluted chromatin of LHON mutant cells and control cells. The difference in histone enrichment between LHON mutant cells and control cells was analyzed using RT‒PCR and statistically determined using the Mann‒Whitney U test (Fig. 6a).

The histone enrichment of *NDUFS4* in LHON fibroblast lines was different from the modifications identified using LHON patient PBMCs. In control fibroblasts, H3K18Ac and H3K4Me3 histone modification enrichment was significantly different (*p < 0.05) from that of LHON mutant cells. In LHON mutant cells, H3K27Ac and H3K27Me3 histone enrichment was significantly different from that of control cells. Other than H3K9Me2 modifications, all other histone modifications showed significant differences in enrichment between the groups (*p* < 0.05) (Fig. 6b).

## Discussion

The precise mechanism of vision loss underlying LHON is extremely difficult to comprehend^24^. Despite the difficulties in clinical translation, we now have to access effective medicines for LHON^3^. The ability to understand varied penetrance, phenotypic heterogeneity and environmental factors causing disorders from this group is a strong incentive to learn more about epigenetics and recognize the importance of epigenetics in the context of mitochondrial diseases^8^. In the present study, we elucidated the effect of histone modifications of *NDUFS4* on LHON pathogenesis. Our findings support the hypothesis that targeting histone modifications of complex 1 subunit-encoding genes would help to better understand the pathophysiology of LHON and the role of epigenetics in LHON.

To sustain proper cellular proliferation and differentiation, epigenetic mechanisms that are well known for their capacity to control the transcription of genes and genomic integrity are essential^22^. Epigenetic processes involve histone modification, microRNAs, DNA methylation and small noncoding RNAs, and these mechanisms are complex events that are not yet completely understood^25,26^. The function of the enzymes that add and remove epigenetic modifications contributes to the maintenance of epigenetic silencing. Development and use of epigenetic tools are a suitable and efficient strategy that can be therapeutically used to the treatment of various diseases, especially with the development of medications that specifically focus on the specific epigenetic mechanisms responsible for the control of gene expression. Pharmacological inhibitors can be used to treat these enzymes to stop their activity^25^. Enzymes that methylate and demethylate H3K4 and H3K27 in the retina have been investigated in loss- and gain-of-function studies, and these studies have revealed the crucial involvement of enzymes in proliferation, differentiation and the timing of differentiation^27^. Studies have discovered that genomic regions may coexist with active H3K4Me3 and repressive H3K27Me3 alterations^25^. In the human retina, the histone modification H3K4Me3 interacts with active promoters. We can thus uncover pertinent regulatory areas crucial for retinal homeostasis and illness by mapping chromatin regions enriched for H3K4me3 and other histone modifications and their chromatin connections^27^.

In our study, we observed enriched expression of H3K4Me3, H3K27Ac and H3K27Me3 in LHON patients compared with healthy individuals. In comparisons of histone modifications between *MT-ND4* mutant cells and control cells, all the specific histone modifications examined in this study, except for the H3K9Me2 modification, showed statistically significant differences in enrichment among the groups. Significant enrichment of histone activation markers of *NDUFS4* in LHON patients is a positive epigenetic regulator that promotes gene expression. Epigenetic regulation might influence the mutation effect of *MT-ND4* to improve the performance of complex 1 in LHON by enhancing the function of the N module and penetrance of the disease. Enhancing the N module function helps improve the electron transfer efficiency, proton pumping capacity and overall stability of complex 1^10^. The actual impact of histone modifications on mitochondrial disease has not been comprehensively studied. Histone modifications associated with nuclear genes, which encode mitochondrial proteins, could indirectly influence mitochondrial function in various ways. In mitochondrial and nuclear genome cross-talk, cellular oxidative stress and metabolic imbalance due to mitochondrial dysfunction affect changes in the patterns of the histone modifications of acetylation and methylation. These changes in patterns are responsible for altered gene expression and amplify disease symptoms^28^. Since the penetrance of LHON is not well understood, more studies are needed to confirm the role of histone modifications in nuclear-encoded mitochondrial proteins in disease penetrance and disease progression. The exact mechanisms behind histone modification variations are not yet known. The current understanding of the mechanisms underlying histone modifications in mitochondrial diseases is still inadequate. Studies have supported that chromatin states are responsible for cell type-specific patterns of gene expression^29^. Numerous environmental factors, such as smoking and alcohol intake, have effects on epigenome modifications in mitochondrial disease^8^. Histone modifications might play a role as an epigenetic factor in LHON that is independent of mitochondrial mutations. Histone modifications are not directly linked to mitochondrial mutations, but it is noteworthy that they could have indirect connections. Mitochondrial dysfunction due to mitochondrial mutations could influence histone modification patterns as part of the regulatory mechanisms. Changes in one process can indirectly influence the other, impacting various cellular functions and contributing to overall cellular health and homeostasis^28^. Our study results indicate that LHON mutations are independent of histone modification differences in *NDUFS4*. Based on the study results, the histone modification differences observed in both LHON-mutant PBMCs and cell lines support the fact that these *MT-ND4* mutations are independent of histone modifications. LHON is considered a complex disease that involves the interaction of various genetic and environmental factors for disease progression.

In conclusion, our study attempted to elucidate the role of epigenetics in LHON through histone modification analysis of the nuclear gene, which encodes the mitochondrial complex 1 protein. Since most mitochondrial proteins are encoded by nuclear genes, epigenetic modifications affect the gene expression and functioning of mitochondria. In addition to primary mutations, other secondary mutations and environmental factors play a role in LHON pathogenesis. To our knowledge, this is the first study to attempt to uncover the histone modifications in *NDUFS4* related to its association with mitochondrial dysfunction specifically in LHON. Furthermore, our findings provide evidence of the overexpression of the activation and repression markers H3K4Me3, H3K18Ac, H3K27Ac and H3K27Me3 in LHON cells compared to control cells. This impacts the complex 1 assembly of *NDUFS4* and might affect the respiratory chain and energy production. A precise interpretation of these results would require further advanced investigations. Thus, these activation and repression markers can serve as epigenetic targets for understanding the role of epigenetics in LHON disease progression and for understanding crosstalk between the mitochondrial and nuclear genomes. Evaluating the histone modifications involved in complex 1 assembly would help to identify the epigenetic markers involved in LHON, and our results will form the basis for further research in epigenetics studies of LHON and future novel approaches to epigenetic-based therapies.
